# Intensity-Modified Recreational Volleyball Training Improves Health Markers and Physical Fitness in 25–55-Year-Old Men

**DOI:** 10.1155/2021/9938344

**Published:** 2021-06-18

**Authors:** Goran Vasić, Nebojša Trajković, Draženka Mačak, Tine Sattler, Peter Krustrup, Nikola Starčević, Goran Sporiš, Špela Bogataj

**Affiliations:** ^1^Faculty of Sport and Physical Education, University of Novi Sad, Novi Sad 21000, Serbia; ^2^Faculty of Sport and Physical Education, University of Niš, Niš 18000, Serbia; ^3^Faculty of Sport, University of Ljubljana, Ljubljana 1000, Slovenia; ^4^Department of Sports Science and Clinical Biomechanics, Faculty of Health Sciences, University of Southern Denmark, Odense 5230, Denmark; ^5^Faculty of Kinesiology, University of Zagreb, Zagreb 10110, Croatia; ^6^Department of Nephrology, University Medical Centre, Ljubljana 1000, Slovenia

## Abstract

The present study is aimed at determining the effects of intensity-modified recreational volleyball training on health markers and physical fitness in healthy middle-aged men. Thirty-four healthy untrained men aged 25–55 years were randomized to either a modified recreational volleyball group (MRV, *n* = 17) or a recreational volleyball group (RV, *n* = 17). Both groups performed volleyball training twice a week over 12 weeks, with participants in MRV playing a modified game with higher intensity due to shorter breaks between rallies. The small to moderate improvements of both groups were observed in SBP (MRV *g*_av_ = −0.50 [-0.67, -0.33] vs. RV *g*_av_ = −0.37 [-0.55, -0.20]) to a similar extent (*p* = 0.12). However, only the MRV significantly improved (*p* < 0.001) the mean body weight (*g*_av_ = −0.35 [-0.52, -0.18]) and BMI (*g*_av_ = −0.39 [-0.56, -0.22]) to a moderate extent and the YYIR1 performance (*g*_av_ = 2.45 [2.22, 2.69]) to a large extent. Even though both groups significantly improved the rest HR, the mean change of rest HR was significantly greater in MRV as compared to the RV (*p* < 0.001,  *ŋ*_**p**_^2^ = 0.47). The study revealed that an intensity-modified type of recreational volleyball, involving shorter breaks between rallies, improves cardiorespiratory fitness and health markers for men aged 25–55 years.

## 1. Introduction

The development of various chronic diseases begins in childhood and adolescence [1–3]. The type of lifestyle we lead therefore determines later quality of life. The importance of physical fitness in promoting quality of life was highlighted in healthy young men [4]. Moreover, it is well documented that cardiorespiratory fitness (CRF) declines with aging in the general population [5] but also among untrained individuals [6]. Considering the fact that low CRF is an important risk factor for cardiovascular and total mortality [7], and that hypertension, diabetes, and hypercholesterolemia as risk factors for cardiovascular disease are influenced by fitness [8, 9], developing exercise programs which is aimed at improving physical fitness should be one of main objectives of national strategies. Additionally, due to the fact that contexts, content, and purposes of PA are the main condition when health or fitness benefits are addressed different types of exercise programs should be introduced and tested [10]. Team sports (games) have been shown to have a positive impact on physical fitness in the adult population [11, 12]. Furthermore, recreational small-sided football in middle-aged men has resulted in improvements in blood pressure, maximum aerobic power and muscle capillarisation, and enhanced fat oxidation [13–18]. Similar findings were observed for the effect of recreational team handball in a study by Hornstrup et al. [19]. The authors discovered that a 12-week intervention led to positive muscular, skeletal, and cardiovascular adaptations, with improved maximal oxygen uptake, lower fat percentage, increased muscle enzymatic activity, and improved bone mineralisation [19]. Significant improvements in cardiovascular and musculoskeletal fitness were found after participation in a small-sided recreational basketball program [20]. However, Trajković et al. found that recreational volleyball did not elicit any changes in cardiovascular fitness in healthy middle-aged men [21]. The reason for this may be that the study was conducted on a full court as 6v6.

Volleyball is an intermittent sport, which means it comprises short, high-intensity bouts followed by lower-intensity actions [21]. However, recreational or small-sided volleyball seems to elicit lower average aerobic exercise intensity compared to football, team handball, and other team sports [22–26]. The reasons why lower HRmax is achieved during volleyball may be the high number of mistakes and excessive standing idle during the game. As some studies suggest that the overall fitness and health effects are higher after exercise interventions with predominantly aerobic high-intensity exercise compared to moderate-intensity exercise [14, 23, 27], it appears relevant to focus on the exercise intensity and the fitness effects of various types of volleyball training. Moreover, exercise with higher intensities evoke higher enjoyment than those with lower intensities [28] which is of great importance in identifying effective and time-efficient exercise modalities that would improve fitness and health status in middle-aged adults.

It would therefore be of interest to determine whether the addition of different actions or changes in rules in a recreational volleyball program would elicit better effects due to increased intensity during the game. Rule modifications in football, such as throwing the ball back to the players, together with encouragement from coaches, have been successful at changing the intensity of the game to a certain level [29]. Moreover, recent study showed that modification in sets during SSG may be important for changing intensities during training [30]. As volleyball has a lot of breaks in the game, the idea was to try to shorten the duration of the breaks by throwing the ball back to the players after rallies. Moreover, given the worldwide popularity of volleyball, there is a need for exercise studies using this game as an intervention for improving health and physical fitness. This study is therefore aimed at determining the effects of intensity-modified recreational volleyball on health markers and physical fitness in healthy middle-aged men. We assumed that improvements in physical fitness and most of the health marker variables will occur following the intensity-modified recreational volleyball intervention.

## 2. Materials and Methods

### 2.1. Study Design

This was a pre-post study that was designed to address the question of how a modification in intensity during volleyball program could affect physical fitness and health markers in middle-aged men. To accomplish this, we screened 22-55 years old men and then randomized them according to a computer-generated sequence. The participants were randomized in a modified recreational volleyball (MRV) and a recreational volleyball (RV) in order to obtain the correct number of participants for recreational volleyball games. Both groups played recreational volleyball over 12 weeks, with MRV playing an intensity-modified game.

### 2.2. Subjects

**Figure 1:**
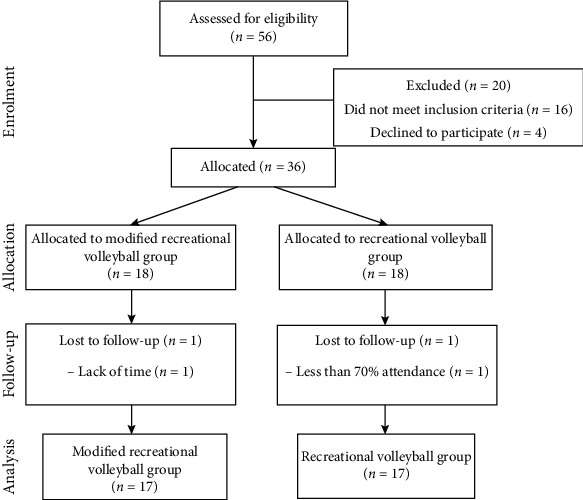
Flow diagram of participant enrolment, randomized group allocation, and final analysis.

Thirty-six healthy untrained men aged 25–55 years agreed to take part in the study after meeting where they were also asked about their history of diseases and medication. The participants were randomly allocated either to a modified recreational volleyball group (MRV, *n* = 17; age, 43.5 ± 5.3 years; height, 182.3 ± 7.3 cm) or a recreational volleyball group (RV, *n* = 17; age, 41.9 ± 5.7 years; height, 183.8 ± 6.4 cm). During the 12-week training program, two subjects withdrew from the study, one due to lack of time and the other due to attending insufficient sessions in the intervention period. Thus, 17 participants remained in each group at final testing (Figure 1). The criteria for inclusion and selection of participants were as follows: male, chronological age 25–55 years, not involved in any type of organised recreational exercise for at least 6 months before the beginning of the program, played volleyball as amateurs and recreationally, and not participating in any other physical exercise program. The criteria for exclusion from the study were as follows: suffering from cardiovascular or respiratory disease, recovering from some form of acute or chronic disease, and in the process of rehabilitation from injury. As all subjects were volunteers, they were able to withdraw from the experimental treatment at any time during the program. Before the beginning of the experimental program, the research and its potential benefits were fully explained to the participants. Additionally, the participants also signed the informed consent statement to take part in the research. The ethical committee approved the study at the Faculty of Sport and Physical Education, University of Novi Sad (Reference No. 46-10-06/2018-5). The study was carried out in accordance with the Declaration of Helsinki.

### 2.3. Procedures

The measurements were performed in the morning in indoor sport hall in the same time and with the same researchers in pre- and posttesting. First, body weight, height, resting heart rate (HR), and blood pressure were measured in a fasting state. Second, participants performed three physical fitness tests in the following order: handgrip strength test, CMJ, and YYIRT1. Participants were asked to refrain from any strenuous activity 48 h prior to all testing and to avoid caffeine 8 h before testing. A standardised warm-up consisting of low intensity running (5 min) and of general exercises such as leg lifts, high skipping, sprints, and lateral running (5 min) was performed before fitness testing. Moreover, a familiarization session was performed for fitness test two days before the testing as well prior to testing.

Body height was measured with a GPM anthropometer (Siber & Hegner, Zurich, Switzerland) to the nearest 0.1 cm. Body weight was measured with a digital scale TANITA BC 540 (TANITA Corp., Arlington Heights, IL) to the nearest 0.01 kg. The following formula was used to calculate body mass index: BMI = body mass (kg)/(height (m)^2^). Upper arm blood pressure monitor (Omron Healthcare, Toronto, Canada) was used to measure blood pressure and resting heart rate. Moreover, the mean arterial blood pressure (MAP = 1/3 SBP + 2/3 DBP) was used for the analyses. The subject had to be placed comfortably in a sitting position in a quiet room, and after 10 minutes of rest, the cuff of the device was placed on the middle part of the left upper arm.

#### 2.3.1. Vertical Jump Performance

Vertical jump performance was tested with countermovement jump (CMJ) without arm swing using an Optojump system (Microgate, Bolzano, Italy). Each participant performed three CMJ repetitions, and the best result, measured in centimeter, was used for further analysis. The validity and reproducibility of vertical jump performance using the Optojump device have proven to be excellent [31, 32]. The CV and the ICC for CMJ were 1.6% and 0.95, respectively.

#### 2.3.2. Yo-Yo Intermittent Recovery Test Level 1 (YYIR1)

Assessment of the cardiovascular fitness was conducted on an indoor basketball court nearly 1 h following the muscular fitness tests. YYIR1 was developed as a tool to determine cardiovascular fitness [33]. YYIR1 consists of 20 m shuttle runs performed at increasing velocities, with 10 s of active recovery between shuttles, until exhaustion. The player stands beside a cone at the starting line. When the audio device beeps, the player runs to another cone at the turning line 20 m away. When the next beep sounds, the player runs back to the starting line. Upon reaching the starting line, the player has a 10 s recovery period, during which he decelerates to another cone 5 m away and walks back to the starting line. When the next beep sounds, the player repeats the shuttles (2 × 20 m). The running speed increases progressively, regulated by the beeps from the audio device. The task is for the player to complete each shuttle before the next beep. The test stops when the player fails to complete a shuttle twice in a row. The result of the test is the total distance covered up to the last completed shuttle. Additionally, to determine HR values, a short-range telemetric heart rate monitor (S 810, Polar Electro Oy, Kempele, Finland) was placed on the players. We used a 5 s interval recording time to monitor heart rate throughout the test. Post hoc HR analyses were performed using the Polar software (Polar Electro Oy, Kempele, Finland). The peak recorded HR was assumed to be the individual's maximal HR [34].

#### 2.3.3. Handgrip Strength

For measuring handgrip strength, a TKK5401 digital dynamometer was used (Takei, Niigata, Japan). The dynamometer measurement was performed with the subject in a standing position, legs spread shoulder-width apart, and arms at the elbows extended along the body. The subject's grip was measured three times with the left hand and three times with the right hand. The results were recorded in kilogram. There was a 1 min resting period between each squeeze to avoid fatigue. The mean value from both hands of the three squeezes was used for further analysis. The CV and ICC for handgrip strength were 1.9% and 0.93, respectively.

#### 2.3.4. Rating of Perceived Exertion (RPE)

Perception of exertion was evaluated using RPE scores on a 10-point scale [35] collected in all training sessions during the training period.

#### 2.3.5. Physical Activity Enjoyment Scale (PACES)

We used the revised version of PACES, which consists of 16 statements [36] scored on a 5-point Likert scale ranging from 1 (disagree a lot) to 5 (agree a lot). A high level of enjoyment of physical activity is indicated when high scores on the positive items and low scores on the negative items are obtained. A total enjoyment score can also be obtained by reversing negative item scores and summing them to positive item scores. With this procedure, total enjoyment scores can range from 16 to 80 (maximum enjoyment). The validity and reliability of PACES were confirmed in adult fitness exercisers [37].

#### 2.3.6. Training Intervention

**Figure 2:**
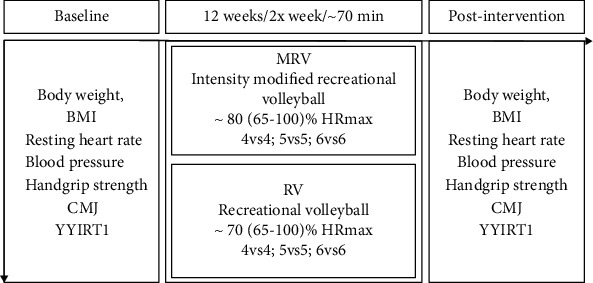
Study design.

The recreational team volleyball training intervention ran for 12 weeks (Figure 2). During this period, both groups performed two training sessions of ~70 min per week with at least 48 h of rest in between. The participants from the experimental group had one week familiarization with training intervention having in mind the modifications of the rules in volleyball game. No explicit feedback or instructions were given. They were provided with the general instructions and the modifications of the rules. Volleyball experts and assistants were involved in familiarization sessions to ensure that there is stability in playing volleyball according to usual and modified rules. The sessions consisted of a standardised 10 min warm-up followed by 60 min of recreational team volleyball matches (4v4, 5v5, and 6v6), interspersed with two 5-minute breaks. The warm-up comprised 5 min of jogging, running at progressively increasing speeds, and 5 min of technical ball drills (passes). The training sessions took place on an indoor volleyball court (18 × 9 m). The average total training attendance over the 12-week intervention period was 21 ± 4 sessions (MRV = 22 ± 3 and RV = 20 ± 4). The difference between the two programs was that MRV had an assistant who delivered the ball to one side of the net or the other after each rally. Having in mind that contacts with the ball are limited in number and duration during volleyball game and that breaks between rallies last from 5.5 to 12 seconds [38], it is of great importance to shorten those breaks, especially during recreational volleyball, where the result is not the primary aim. Moreover, the involvement and encouragement of the assistant were shown to induce the game intensity [39]. The participants in the RV group played a usual volleyball match, with serving after each rally. There was a familiarization session with participants being familiarized with the change of including assistant that throw balls instead of serving. The participants from both groups were asked not to change their usual diet or habitual physical activity apart from the intervention. Heart rate monitoring during sessions was performed using a Polar heart rate monitor (S 810, Polar Electro Oy, Kempele, Finland) once a week. As stated earlier, for all participants, maximal HR was calculated by an YYIRT1 assessment test and based on that the load was determined. Moreover, participants reported RPE and enjoyment immediately after each game.

#### 2.3.7. Statistical Analysis

Data are presented as mean ± SD unless otherwise stated. The G^∗^power 3.1 power analysis software determined the minimum sample size (*N* = 22) given the critical *F* = 4.35, an effect size *f* = 0.32 (*ŋ*_*p*_^2^ = 0.09), *p* = 0.05, 1 − *β* = 0.8, groups and time points = 2, and corr = 0.5. Data are presented as mean ± SD unless otherwise stated. Residuals were normally distributed as confirmed by a Shapiro-Wilk test and a visual inspection of histogram. The Levene's and Box's tests failed to reject homogeneity of variances and covariance matrices, respectively. A *t*-test for independent samples determined whether baseline group differences in study outcomes occurred and did the training intensity, RPE, and PACES differ among the groups. A 2 (MRV vs.RV) × 2 (pre vs. posttest) mixed ANOVA evaluated the effects of playing modified recreational volleyball on the study outcomes in respect to traditional recreational volleyball after twelve weeks. Given a treatment^∗^time interaction effect, we inspected the study outcome mean changes with 95% confidence intervals (95% CIs) from baseline to after 12 weeks depend on whether subjects received the MRV or RV. We consequently estimated a simple main effect of time analyzing mean changes from baseline to after 12 weeks separately for each group with a Bonferroni adjusted *p* values and 95% CIs. Partial eta squared (*ŋ*_*p*_^2^) is reported as the effect size measure for the interaction effects and classified as small (0.01), moderate (0.06), and large (0.14) [40]. The Hedges's *g*_av_ with 95% CIs designated the size of simple main effect of time and interpreted as small (±0.20), moderate (±0.50), and large (±0.8). The level of significance was set at *p* ≤ 0.05. All statistical analyses were performed in the SPSS statistical software (SPSS 23.0, IBM Inc., Chicago, IL, USA).

## 3. Results

### 3.1. Training Intensity

**Figure 3:**
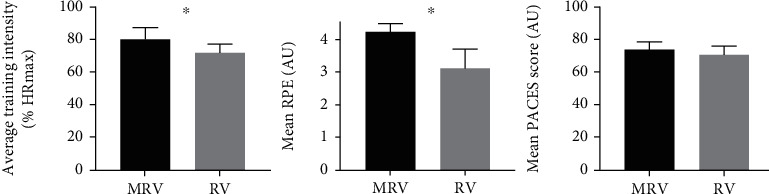
Mean heart rate, mean RPE, and mean PACES score in MRV and RV during the intervention. Means ± SD are presented. Abbreviations: AU: arbitrary units; MRV: modified recreational volleyball group; PACES: physical activity enjoyment scale; RV: recreational volleyball group. ^∗^*p* < 0.05 significant differences between MRV and RV.

**Figure 4:**
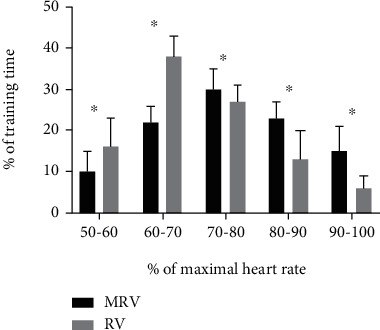
Time spent (% of training time) in various heart rate zones as percentage of maximum heart rate (HRmax) during modified recreational volleyball (MRV, black bars) and recreational volleyball (RV, grey bars). Data are presented as means ± SD. ^∗^*p* < 0.05 significant differences between MRV and RV.

Average training intensity was 80 ± 7% HRmax for MRV compared to 72 ± 7% HRmax, respectively, for RV (*p* < 0.05; Figure 3). HR distribution in relation to the percentage of training time in target HR zones is presented in Figure 3. Participants spent more time in the heart rate zone 80–90% (*p* < 0.05) during MRV than during RV (23.1 ± 4.3% versus 13.2 ± 7.2%), as well as above 90% (*p* < 0.05; 15.5 ± 6.2% versus 6.4 ± 3.2%) (Figure 4). Additionally, average RPE for MRV was 4.25 ± 0.24 compared to 3.14 ± 0.24 for RV. MRV showed a higher score on the PACES enjoyment questionnaire compared to RV (73.7 ± 4.6 vs. 70.9 ± 5.2), but without statistically significant differences.

### 3.2. Comparison of Modified (MRV) and Traditional Recreational Volleyball (RV) Effects on Study Outcomes

Baseline body weight (*p* = 0.76), BMI (*p* = 0.43), rest HR (*p* = 0.50), SBP (*p* = 0.65), DBP (*p* = 0.82), MAP (*p* = 0.73), YYIR1 (*p* = 0.92), handgrip (*p* = 0.74), and CMJ (*p* = 0.69) were similar between the MRV and RV.

**Figure 5:**
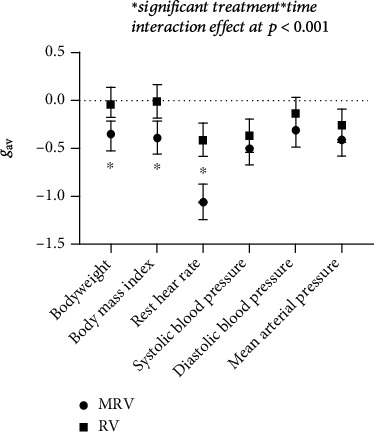
Hedges's *g*_av_ with 95% CIs on health markers.

**Figure 6:**
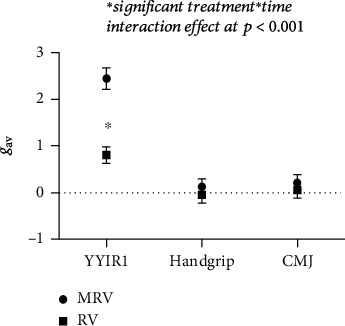
Hedges's *g*_av_ with 95% CIs on performance of Yo-Yo intermittent recovery test level 1 (YYIR1), handgrip, and countermovement jump (CMJ).

The mean body weight and BMI significantly decreased to a moderate extent only in the MRV (Figure 5). The average YYIR1 performance also significantly increased to a large extent only in the MRV (Figure 6). Even though both groups significantly improved rest HR, the mean change of rest HR was significantly greater in MRV than in the RV. However, the small to moderate improvements of SBP were observed in both groups. There were no significant changes in either group for handgrip strength (*p* = 0.60) or CMJ (*p* = 0.15) after the 12-week intervention.  Table 1 shows the detailed results from the 2 × 2 ANOVA.

## 4. Discussion

This study is aimed at comparing the effects of modified recreational volleyball and regular recreational volleyball on physical fitness and health markers in healthy middle-aged men. The findings showed that 12 weeks of modified recreational volleyball with higher exercise intensity and higher perceived fun improved cardiorespiratory fitness and decreased some risk factors, specifically resting HR, body weight, and BMI, compared to regular recreational volleyball. These findings provide support to the hypothesis that the exercise intensity is of importance for the physical fitness and health outcomes of recreational volleyball training.

The YYIR1 test showed good criterion validity comparing to laboratory VO_2_max in recreationally active subjects (*r* = 0.87) with the reported coefficients of variation of 8.7% [41]. Recent systematic review [42] stated that YYIRT reference values differ regarding the type and level of sport performed. The results in the current study (posttest: 1064 ± 92 m) are somewhat lower compared to reference values from 211 recreationally active adults (1339 ± 53 m). However, there were no participants that were engaged in recreational volleyball which makes comparison difficult [42]. Nevertheless, the YYIR1 performance had increased markedly in MRV (18.7%) compared to RV (3.3%) after the 12-week intervention (*g*_av_ = 2.45 [2.22, 2.69], large ES). A similar study found only a 2.4% change (8 m) in YYIR2 performance following 10 weeks of small-sided recreational volleyball practice [43]. Another study [22] conducted on a full court showed that the recreational volleyball group improved shuttle run test performance by 4.3% between pre- and posttests, indicating a small increase in VO_2_max, while a 3.2% decrease was observed in the control group. Bigger improvements in cardiovascular fitness following modified recreational volleyball training compared to recreational volleyball were mainly due to higher intensity in MRV (80% HRmax versus 72% HRmax) as well as more time spent in higher zones (Figure 3). The higher intensity in modified training/preparatory games compared to regular game conditions has been confirmed in professional volleyball [44]. Recent study showed that the involvement and encouragement of the assistant were shown to change the game intensity [39]. This was confirmed in the current study, where the higher intensity was obtained during a modified volleyball game (Figure 2).

However, besides higher intensity in MRV, the achieved YYIR1 performance change was lower than reported in recreational handball (80%) [25] and recreational football (37–49%) [17] after a similar intervention duration and with similar participants. A recent meta-analysis [17] showed that the intensity of 78–84% HRmax in recreational football is sufficient for the 8–13% improvement in VO_2_max in healthy untrained men. The results of the current study for cardiorespiratory fitness therefore revealed that MRV represents a good stimulus for significant improvements after 12 weeks of intervention in healthy middle-aged men.

By contrast, we found no significant changes for countermovement jump performance and handgrip strength after 12 weeks of MRV, with only small improvements compared to RV. As volleyball requires explosive jumps and fast-paced actions [45], it was expected that changes in strength in our study would be somewhat greater. However, the volume of jumps and game intensity in volleyball increase at higher levels [32]. It could be assumed that the great majority of jumps during both types of recreational volleyball were submaximal, which may have impacted on our results. Nevertheless, the CMJ results in the current study revealed a tendency for improvement in MRV (+6.2%) compared to RV (+1.7%), albeit these changes did not reach significance. Another possible reason for the small improvements could be the duration of the program. Several studies have shown that longer interventions elicit better improvements in jump performance [13, 46, 47].

The results showed a practical small (+1.2%) improvement in handgrip strength in MRV after 12 weeks. Similar improvements (+3%) were found after recreational handball training in 33–55-year-old sedentary males. Further investigations are warranted, given the importance of handgrip strength and its association with increased risk of cardiovascular and all-cause mortality [48].

Positive effects on cardiovascular risk factors such as resting HR, body weight, and BMI were observed after 12 weeks of modified recreational volleyball. The MRV significantly improved the mean body weight (moderate ES = −0.35 [-0.52, -0.18]) and BMI (moderate ES = −0.39 [-0.56, -0.22]). Resting HR is used as an independent noninvasive predictor of cardiovascular diseases [49, 50], since the risk of such illnesses rises with an increase in resting HR above 60 beats per minute (bpm) [51]. The baseline values in our study were higher than 60 bpm, meaning that the more pronounced drop in resting heart rate for the MRV group, may well be of importance for the overall health profile.

Mean diastolic and systolic blood pressure were lowered after the intervention in both groups, with no significant between-group differences. Previous studies have demonstrated that recreational football, handball, floorball, and volleyball successfully decrease blood pressure [13, 19, 22, 52]. Furthermore, the participants in the present study showed higher baseline blood pressure values, so further reductions would be of significant importance bearing in mind that values from 115/75 mmHg increase the risk for cardiovascular diseases [53].

Obesity represents a risk factor for a number of chronic diseases. Our participants had baseline BMI values just above normal (25 kg/m^2^) and can therefore be considered overweight. Hence, a lowering of body weight would be needed to improve health profile. After the intervention period, the participants in MRV had lowered their BMI values, presumably because they were more active and had higher exercise intensity (Figure 2).

The fact that physical activity and nutrition were not fully controlled could be stated as study limitation. This might have affected the training effect on some health markers. However, the participants stated that they had their usual diet and were not engaged in any other organised physical activity programs. Moreover, the present study did not use treadmill measurements to evaluate cardiorespiratory fitness, which is considered as important limitation as the results could not be compared with VO_2_max reference values. Despite the interest in extending recreational football benefits to other team sports, only two studies [22, 26] involving recreational volleyball have been published in the last five years. These were conducted in order to explore the potential of other team sports in an attempt to find other novel exercise modes that would be as effective as recreational football. However, the results of the abovementioned studies showed that the intensity of recreational volleyball was not high enough to detect significant changes in cardiorespiratory fitness. The current study showed that modified recreational volleyball, with its higher intensity, significantly enhanced cardiorespiratory fitness in middle-aged men. Playing our favourite team sport twice a week for ~1 h with friends therefore has numerous health and social benefits. Another positive factor that could encourage people to engage in recreational volleyball is that it can be played with 4–12 players with no particular demands in respect of facilities, organisation, and a group of committed participants.

## 5. Conclusions

In summary, intensity-modified recreational volleyball training for men aged 25–55 years has a positive impact on physical fitness and health markers. Moreover, the men playing the modified volleyball game also had high enjoyment scores, emphasizing that the modified version of recreational volleyball appears to be a good tool to optimize the physiological and psychological benefits of volleyball.

## Figures and Tables

**Table 1:** Comparison of study outcomes among the groups playing modified recreational volleyball (MRV; n=17) and traditional recreational volleyball (RV; n=17) at baseline and after 12 weeks.

Group	Pre-test	Post-test	Mean change [95% CIs]	A2x2 mixed ANOVA: group-by-time interaction effect			
				F_(1, 32)_	p	*ŋ*_*p*_^2^	1-*β*
Bodyweight^¥^ (kg)							
MRV	86.33 ± 6.27	84.08 ± 6.04	−2.50 (−2.90,−1.60)^∗∗^	22.11	<0.001	0.50	0.99
RV	87.00 ± 4.31	86.83 ± 4.15	-0.17 (-0.28, -0.48)				
BMI^¥^ (kg/m^2^)							
MRV	26.42 ± 1.63	25.73 ± 1.77	−0.69 (−0.88,−0.50)^∗∗^	26.27	<0.001	0.54	0.99
RV	25.90 ± 1.50	25.88 ± 1.48	-0.03 (-0.22, 0.16)				
Rest HR^¥^ (bpm)							
MRV	67.83 ± 3.38	64.42 ± 2.84	−3.42 (−4.17,−2.67)^∗∗^	19.32	<0.001	0.47	0.99
RV	68.67 ± 2.57	67.50 ± 2.11	−1.17 (−1.92,−0.42)^∗∗^				
SBP^¥^ (mmHg)							
MRV	133.08 ± 10.16	128.50 ± 7.62	−4.58 (−6.49,−2.67)^∗∗^	2.56	0.12	0.10	0.33
RV	134.67 ± 6.54	132.17 ± 6.60	−2.50 (−4.41,−0.59)^∗∗^				
DBP^¥^ (mmHg)							
MRV	86.67 ± 4.42	85.25 ± 4.48	−1.42 (−2.47,−0.37)^∗∗^	1.35	0.26	0.06	0.20
RV	87.08 ± 4.38	86.50 ± 3.78	-0.58 (-1.64, 0.47)				
MAP^¥^ (mmHg)							
MRV	102.10 ± 6.11	99.72 ± 5.20	-2.38 (-7.20, 2.20)	3.35	0.08	0.13	0.42
RV	102.89 ± 4.69	101.71 ± 3.97	-1.18 (-4.86, 2.50)				
YYIR1 (m)							
MRV	896.00 ± 40.00	1064.00 ± 92.00	138.00 (128.00,206.00)^∗∗^	26.19	<0.001	0.58	0.99
RV	898.00 ± 27.00	928.00 ± 44.00	30.00 (-11.00, 71.00)				
Handgrip (kg)							
MRV	51.82 ± 4.60	52.45 ± 4.61	0.64 (-0.56, 1.83)	1.02	0.33	0.05	0.16
RV	52.50 ± 4.70	52.30 ± 4.52	-0.20 (-1.45, 1.05)				
CMJ (cm)							
MRV	33.05 ± 9.87	35.11 ± 8.97	2.06 (-0.52, 4.65)	0.67	0.42	0.03	0.12
RV	34.75 ± 9.06	35.35 ± 9.30	0.60 (-2.11, 3.31)				

Values are Mean±SD. Abbreviations: ^¥^ reverse scoring; BMI, body mass index; HR, heart rate; SBP, systolic blood pressure; DBP, diastolic blood pressure; MAP, mean arterial pressure; YYIR1, Yo-Yo intermittent recovery test level 1; CMJ, countermovement jump; mean change [95% CIs], mean difference from pre- to post-tests with 95% confidence intervals; F(df_factor_, df_error_), F-statistic; p, p value; *ŋ*_*p*_^2^, partial eta squared; 1-*β*, post-hoc statistical power of the test; ^∗∗^ significant pre-to post-tests change at p<0.01; ^∗^ significant pre-to post change at p<0.05.

## Data Availability

Data available on request.
